# The effect of acute respiratory events and respiratory stimulants on EEG-recorded brain activity in neonates: A systematic review

**DOI:** 10.1016/j.cnp.2023.11.002

**Published:** 2023-11-10

**Authors:** Fatima Usman, Simon Marchant, Luke Baxter, Hamisu M. Salihu, Muktar H. Aliyu, Eleri Adams, Caroline Hartley

**Affiliations:** a Department of Paediatrics, University of Oxford, Oxford, UK; b Kano Independent Research Center Trust, Kano, Nigeria; c Department of Health Policy and Vanderbilt Institute for Global Health, Vanderbilt University Medical Center, Nashville, TN, USA; d Newborn Care Unit, Oxford University Hospitals NHS Foundation Trust, Oxford, UK

**Keywords:** EEG, Preterm, Inter-breath interval, Respiration, Respiratory stimulants, Apnoea

## Abstract

**Objective:**

We conducted a systematic review to investigate electroencephalography (EEG) changes during periods of acute respiratory events such as apnoea and the effect of respiratory stimulants on EEG features in infants.

**Methods:**

Studies examining respiration and EEG-recorded brain activity in human neonates between 28 and 42 weeks postmenstrual age were included. Two reviewers independently screened all records and included studies were assessed using the Joanna Briggs Institute Critical Appraisal Tool. The protocol was registered in PROSPERO (CRD42022339873).

**Results:**

We identified 14 studies with a total of 534 infants. Nine articles assessed EEG changes in relation to apnoea, one assessed hiccups, and four investigated the effect of respiratory stimulants. The relationship between neonatal apnoea and EEG changes was inconsistent; EEG suppression and decreased amplitude and frequency were observed during some, but not all, apnoeas. Respiratory stimulants increased EEG continuity compared with before use.

**Conclusions:**

Current studies in this area are constrained by small sample sizes. Diverse exposure definitions and outcome measures impact inference.

**Significance:**

This review highlights the need for further work; understanding the relationship between respiration and the developing brain is key to mitigating the long-term effects of apnoea.

## Highlights

• Few studies investigated EEG changes during apnoea and in relation to respiratory stimulants in neonates.
• EEG suppression is observed during some apnoeas but diverse definitions of apnoea and EEG measures limit inference.
• Respiratory stimulants increase EEG continuity compared with before use.

## 1 Introduction

Neonates, especially those born prematurely, have irregular breathing patterns with frequent pauses in their breathing – a manifestation of the developmental immaturity of brain respiratory control mechanisms (Abu-Shaweesh and Martin, 2008, Picone et al., 2014, Williamson et al., 2021). Apnoea – often defined as a pause in breathing of at least 20 s, or shorter if accompanied by bradycardia or cyanosis (Committee on Fetus and Newborn. American Academy of Pediatrics, , 2003, Elder et al., 2013) – is one of the commonest neonatal respiratory emergencies, and occurs frequently in very preterm infants (Eichenwald, 2016, Pergolizzi et al., 2022), with the risk extending beyond term gestation in some infants (Eichenwald et al., 1997). Moreover, respiratory conditions are a common reason for admission to the neonatal unit, for both term and preterm infants (Gallacher et al., 2016). Indeed, the term infant, despite having a relatively mature central nervous system (CNS) may present with acute respiratory events including apnoea (Levin et al., 2017) secondary to CNS related complications, metabolic, infectious or obstructive causes (Patrinos and Martin, 2017).

As the neonatal brain is developing, it is vulnerable to hypoxic insults, and pauses in respiration can impair cerebral perfusion and oxygenation (Choi et al., 2021, Horne et al., 2018, Martin et al., 2011). Apnoea and hypoxia have been associated with long-term effects, including poor neurodevelopmental outcomes (Janvier et al., 2004, Pillekamp et al., 2007, Poets et al., 2015). In contrast, pharmacological therapies including caffeine, aminophylline and doxapram (routinely used in the Newborn Intensive Care Unit either prophylactically or for treatment of apnoea of prematurity), can reduce the incidence of apnoea (Henderson-Smart and De Paoli, 2010) and the risk of adverse neurocognitive outcomes (Schmidt et al., 2007). However, these studies do not necessarily show that apnoea has a causal association with neurodevelopmental outcomes (Williamson et al., 2021).

Key to understanding the potentially cyclical relationship between brain development and apnoea is to identify how changes in respiration impact brain function, and to compare changes in brain activity during apnoea, with changes in brain activity during periods of other acute respiratory events (such as shallow breathing) (Williamson et al., 2021). Moreover, investigating changes in brain function with the use of respiratory stimulants will enable a greater understanding of how such interventions potentially mitigate the effects of apnoea on the developing brain and their neuroprotective function. Brain activity is essential for development – in animal models, blocking or reducing activity during critical periods of development leads to abnormal, weak, thalamocortical connectivity and a lack of structure within the cortex (Ghosh and Shatz, 1992, Kanold et al., 2003, Tolner et al., 2012). Altering the pattern of activity in a computational model of the developing cortex disrupts synaptic connectivity formation (Hartley et al., 2020). In human neonates, reduced cortical activity early in life is associated with poor neurodevelopmental outcomes (Ranasinghe et al., 2015, Wikström et al., 2012). Understanding whether and how brain activity is altered during apnoea, how this compares with brain activity changes during other respiratory events, and how brain activity is altered by respiratory stimulants will help elucidate the mechanism by which apnoea impacts the developing brain.

We conducted a systematic review to determine the current understanding of how brain activity recorded using EEG changes during periods of acute respiratory events (e.g., apnoea, periodic breathing, shallow breathing, tachypnoea, bradypnea, etc.) and with the use of respiratory stimulants in human neonates between 28 and 42 weeks postmenstrual age (PMA). Specifically, we aimed to identify the current knowledge regarding (1) how respiratory and EEG signals co-vary in neonates across different PMA during normal breathing, (2) the differences in the EEG signals of neonates between periods of normal respiration and periods of acute respiratory events, (3) the relationship between the characteristics of acute respiratory events (e.g., duration, degree of severity) and brain activity changes, and (4) the effect, if any, of respiratory stimulants such as caffeine citrate, doxapram, or aminophylline on EEG features.

## 2 Methods

This systematic review is reported in accordance with the Preferred Reporting Items for Systematic Reviews and Meta-Analyses (PRISMA) statement (Page et al., 2021). The protocol for this systematic review was registered in PROSPERO (CRD42022339873) (Usman et al., 2022).

### 2.1 Exposure and comparison group

The exposure variables were defined as any abnormal, irregular, or dysfunctional acute respiratory event such as apnoea, periodic breathing, tachypnoea, bradypnea, sighing; and any breathing pattern such as shallow breathing, hyperpnea or respiratory-related muscle contractions, like hiccups. Geographical and institutional variations in the definition of operational terms exist and any article with a clear description of the exposure variable was included.

We considered within-subject comparison with a period of normal respiratory recording, (i.e., respiratory-event-free period), or a between-subject comparison with a neonatal cohort having normal respiratory rate, (i.e., the average rate between 40 and 60 breaths per minute and not experiencing any abnormal respiratory event) as a valid comparison for any exposure.

### 2.2 Review inclusion and exclusion criteria

All study designs reporting on acute respiratory events or the use of respiratory stimulants and EEG-recorded brain activity in human neonates between 28 and 42 weeks postmenstrual age (PMA) were included. We excluded the following: review articles, systematic reviews, commentaries, questionnaires, survey reports, and studies with wrong study population (animals, non-neonatal e.g., adults or older children). We also excluded studies involving neonates with cardiovascular malformations; neurological abnormalities (e.g., central nervous system malformations, seizures, hypoxic-ischemic encephalopathy), and intraventricular haemorrhage. Additionally, for the comparison group we excluded studies that involved neonates with pneumonia, bronchiolitis, congenital respiratory malformations or any abnormal respiratory event. Finally, we excluded studies that used EEG recordings for sleep staging only.

### 2.3 Search strategy

We used a combination of Medical Subject Headings (MeSH) terms and controlled keywords including three subject domains, namely EEG, breathing and infant categories; and an appropriate database-specific search strategy across eight databases (MEDLINE, Embase, Global Health, PsycINFO, Cumulative Index of Nursing and Allied Health Literature, Cochrane Library, Science Citation Index and Conference Proceedings Citation Index via Web of Science, and the grey literature on ProQuest). All search strategies are provided in full in Appendix A. Only studies reported in English were included with no publication year restriction up to 2022. The initial search was conducted in January 2022 and updated in August 2022 to capture recent publications. We also performed a backward citation search of all included articles.

### 2.4 Study selection

Article selection was performed using Rayyan software for systematic reviews (Ouzzani et al., 2016) based on the prespecified inclusion and exclusion criteria. Following de-duplication on Rayyan of the uploaded articles from the search results, initial title and abstract screening was done by two reviewers (F.U., S.M.). Before formal screening commenced, piloting of the selection process on a subset of 20 randomly selected articles against the set inclusion and exclusion criteria was performed by the two reviewers and the arbitrator to ensure the selection criteria were applied consistently. Each reviewer independently screened the articles for inclusion and was blinded to the decision by the other reviewer. At the end of the first selection process, the reviewers’ decisions were compared, and discrepancies were resolved through discussion. Where differences still existed after discussion, a third reviewer (C.H.) acted as an arbitrator. Following title and abstract screening, full-text screening was conducted of the remaining articles independently by the same two reviewers. The arbitrator resolved any discrepancies. The reason for exclusion was documented for each ineligible article.

### 2.5 Data extraction

Review-specific data extraction fields were developed, piloted, and refined before final data extraction. Information was transcribed onto an excel sheet including the first author’s name, journal title, year, country, and World Health Organisation region of study; study period, study design, sample size, age (gestational, postmenstrual or postnatal), infant category (term or preterm) and sub-population (e.g. infants with low-birth weight) of infants studied (where specified); type of exposure variable, including definition, comparison group (where stated), type of respiratory stimulant used (if any); EEG type (conventional or amplitude-integrated), montage, number of channels, corresponding EEG findings; and cardiovascular monitoring where reported. No authors were contacted because the selected articles detailed all relevant information.

### 2.6 Outcome measures description

The primary outcomes were any EEG feature and changes thereof recorded using conventional and amplitude-integrated EEG and related to neonatal respiration, namely (but not limited to):

1. Band power (absolute and relative band powers of the full EEG signal in filtered bandpass range, and the delta, theta, alpha and beta bands);
2. Frequencies (average frequency of the full EEG signal; and in the delta, theta, alpha and beta band, 90 % spectral edge frequency);
3. Amplitudes (minimum, maximum and average absolute amplitude in the delta, theta, alpha and beta bands);
4. Continuity pattern (inter-burst interval duration, degree of continuity, occurrence of burst suppression patterns);
5. Entropy (multiscale entropy, approximate entropy, sample entropy, power spectral entropy).

### 2.7 Risk of bias (Quality) assessment

Two reviewers (F.U., C.H.) independently evaluated the risk of bias for the included studies using the Joanna Briggs Institute (JBI) critical appraisal tool for systematic reviews (Aromataris and Munn, 2020). This tool was chosen as it offers a range of checklists for different study designs. Each study-specific appraisal checklist had the options of “yes”, “no”, “unclear”, or “not applicable” – discrepancies between reviewers were resolved through discussion. Due to the low number of studies identified for inclusion in the review, we used the JBI tools to assess the quality of conduct and reporting and not to make exclusion decisions for the included articles. Checklist results are given for all included studies in Appendix B.

### 2.8 Data synthesis

A PRISMA flowchart was used to depict the outcome of the database searches and the selection process. A narrative approach described by the Centre for Reviews and Dissemination for systematic reviews (Centre for Reviews and Dissemination, 2009, Popay et al., 2006) was used to summarise all empirical evidence detailing the relationship between respiratory changes and EEG features. Details for all included articles were summarised in tables. A thematic description of the study findings was discussed while exploring variations in baseline characteristics of the study population, methodology, and differences in exposure and outcome measures. During narrative synthesis, three distinct themes were identified in the articles identified – those investigating EEG changes during apnoea, those investigating EEG changes during other respiratory events, and those investigating EEG changes to respiratory stimulants; these themes are described separately in the results. Finally, the limitations and strengths of the review were explored using the quality and data completeness of the included studies.

## 3 Results

**Fig. 1:**
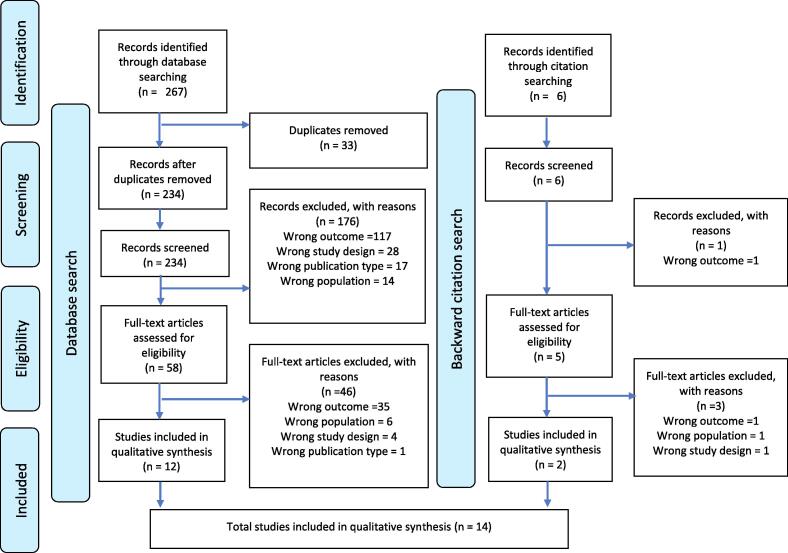
Prisma flow chart showing search outcome and article selection process for the review.

The electronic databases search identified 234 relevant articles after removing 33 duplicates. Most (n = 176, 75.2 %) were excluded following the title and abstract review, and a further 46 (19.6 %) were excluded following the full-text screen. Overall, 14 articles comprising a total of 537 infants were included in the review synthesis (Fig. 1). Half of the studies (n = 7/14, 50 %) involved preterm infants only – with one study focusing on low-birthweight infants, while one (7.1 %) study included term infants only. Most of the publications were from Europe (n = 9/14, 64.3 %), almost a quarter from the region of the Americas (n = 3/14, 21.4 %) and one each from the Eastern Mediterranean and Western Pacific regions (n = 1/14, 6.3 %, each). No studies were reported from Africa. Only one study (7.1 %) was an RCT while the others were descriptive in design (cross-sectional, n = 8/14, 57.1 %; cohort studies, n = 4/14, 28.6 %; or case reports, n = 1/14, 7.1 %). Two studies were reported as conference abstracts only.

Most studies (n = 9/14, 64.3 %) focused primarily on the relationship between apnoea and EEG activity, one (7.1 %) reported other respiratory changes (hiccups) while just over a quarter (n = 4/14, 28.6 %) investigated the effect of respiratory stimulants on EEG features. We did not identify any study that examined how normal respiration and EEG signals co-vary in neonates. The EEG measures evaluated were diverse (Table 1, Table 2, Table 3). Conventional EEG (cEEG) was used in 9 studies (64.3 %), amplitude-integrated EEG (aEEG) in 3 studies (21.4 %), one (7.1 %) study used both aEEG and cEEG, and in one study (7.1 %) the EEG method was not specified (this was a conference abstract). A bipolar EEG montage was used during most (n = 10/14, 71.4 %) recordings. Of note, aEEG was used in all 4 studies investigating the effect of respiratory stimulants (Table 3).

**Table 1:** Summary of included studies reporting the effect of apnoea on neonatal EEG features.

**Author (year)**	**Country and WHO Region**	**Study design**	**Infant age (weeks)**	**Sample size**	**Comparison group**	**Respiratory and cardiovascular changes definition**	**EEG methods**	**Results**	**Quality assessment/Comments**
Deuel (1973)	USA, America.	Cross-sectional.	Term and preterm (32–39 GA, 2–8 PNA).	13: 10 with eligible population inclusion criteria for this review (3 excluded as 2 had GA of 52 and 58 weeks and 1 had apnoeic seizures).	Within and between subjects; during regular period of respiration.	Apnoea: cessation of breathing > 20 s measured using impedance pneumography. Respiratory pauses: intervals > 2 s and < 20 s with no thoracic wall movement. All subjects had ECG recorded and bradycardia defined as any R-R interval of 0.6 s or greater. Oxygen saturation was not recorded	Montage: At least 4 channels bipolar cEEG (Fp1-C3, Fp2-C4, C3-O1, C4-O2). Outcome measures: Degree of continuity and burst suppression pattern. Quantification/ assessment methods used were unclear. Sleep states were differentiated into active (movement on EOG, movement artifact on EEG, observer comment), quiet (no evidence of movement on EOG or EEG), wakefulness (20 sec epoch with eyes opened or crying noted)	3 neonates had apnoea. 1 neonate met this review's inclusion criteria. They had 4 apnoeas commencing during quiet sleep and manifesting as simultaneous apnoea and EEG suppression. The initial EEG suppression was no longer than suppression during regular respirations in this patient. Bradycardia occurred approximately 15 s after apnoea and EEG suppression were observed in the detailed example given. All 10 infants had pauses in respiration (2–20 secs). Of pauses in active sleep, the greater proportion (80 %) were between two and five seconds in duration, whereas of pauses in quiet sleep, the greater proportion (54 %) were between 5 and 20 s in duration. Bradycardia occurred concomitantly. Frequently the start of a respiratory pause coincided with a burst suppression pattern, but this was not always true. The author does not give the frequency of these changes or in which sleep state they occurred.	No clear inclusion criteria and little information on subject demographics. Recording methods clearly described, but visually assessed to identify pauses in breathing and EEG changes. No statistical analysis.
Fenichel et al. (1980)	USA, America.	Cross-sectional.	Term and preterm (≤38 PMA).	15: 11 infants had nonconvulsive apnoea and met the inclusion criteria for this review.	Within-subject; before, during and after apnoea.	Apnoea: ≥4 s, measured using a thermocouple taped under the patient’s nose in non-intubated infants, or a piezoelectric transducer on the abdomen in intubated infants. Heart rate changes were measured using ECG and described as a percentage decrease from baseline.	Montage: 3 different 6 channel bipolar cEEG montages were used (Fp1-T3, Fp2-T4, Cz-C3, Cz-C4, T3-O1, T4-O2; Fp1-Fp2, Cz-C3, C3-T3, Cz-C4, C4-T4, O1-O2; Fp1-C3, Fp2-C4, C3-O1, C4-O2, O1-T3, O2-T4) Outcome measures: Visual assessment of EEG pattern specifically amplitude and frequency changes and sleep states. Active sleep was defined by movement on EOG and EMG with decreased tone, continuous EEG and irregular breathing while the opposite was described as quiet sleep. Any state that did not meet the criteria above was considered as indeterminate sleep.	35 apnoeas were observed in the 11 infants. 19 episodes were ≤ 19 s while 16 were ≥ 20 s. Of the 19 apnoeas ≤ 19 s (10 occurred in active, 5 in quiet, while 4 were in indeterminate sleep), 6 showed mild amplitude suppression, while 13 had no observed changes. Likewise, heart rate changes were inconsistent, showing an increase in 4, a decrease in 12 (with a median time to change of 2 s and return to pre-apnoea level within 6 s) and unchanged heart rate in 3 episodes. Of the 16 apnoeas ≥ 20 s (3 in active, 8 in quiet and 5 in indeterminate sleep), only 1 episode showed mild EEG amplitude suppression. No EEG change was observed in the other episodes. Heart rate decreased in all but 1 apnoeic episode which also had no EEG change. The median time to bradycardia was 3 s and return to baseline within 22 s)*.*	Inclusion/exclusion criteria were clearly defined, and methods of recording EEG and apnoea were clear. Definition of apnoea used is not standard. Outcome assessment was visually performed limiting reporting validity and reliability. No statistical analysis was performed.
Bridgers et al. (1985)	USA, America.	Cross-sectional.	Term and preterm (Grouped into to < 30, 30–33, 34–37 and ≥ 38 weeks GA).	50: 38 met eligibility criteria (12 had seizure diagnosis independent of apnoeic episodes, treated with antiepileptics during recording). 37 of the 50 infants had apnoeic episodes during the EEG recording – 10 of these had seizure diagnosis but results not reported separately for this group.	Within and between subject; before during and after apnoea.	Apnoea: ≥15 s with or without bradycardia (≤90 beats per minute) using monitor alarms and identified by an event marker and nursing log entry. ECG was recorded in 27 infants and bradycardia defined as heart rate < 90/minute. Oxygen saturation measurements were not described.	Montage: 3 channels bipolar cEEG (P3-F3, T3-T4. F4-P4). Outcome measure: Visual assessment of amplitude. Sleep states were not described.	153 apnoeas were detected. Loss of EEG amplitude was observed during some, but not all apnoeic episodes. However, the authors do not specify the frequency of amplitude suppression and heart rate changes during apnoeic episodes. No apnoeas were associated with seizure activity on the EEG.	The main aim of the paper was to determine whether apnoeas were related to electrical seizures. Only brief mention was given to EEG changes associated with apnoea. This was based on subjective visual assessment of 2 raters. No statistical analysis was conducted.
Wulbrand and Bentele (1994)	Germany, Europe.	Cohort-conference abstract.	Preterm (mean GA 28 weeks range 26.1 to 32.1 weeks, recorded at 36, 40, 44 and 52 weeks)	10	Within-subject, at 36, 40, 44 and 52 weeks conceptional age.	Apnoea:>10 s assessed using nasal airflow and chest wall movement. Augmented breath or sigh (not defined). ECG was recorded but bradycardia definition and oxygens saturation measures were not specified.	Montage: Not specified. Outcome measure: EEG suppression. Quantification/ assessment methods used were unclear. Sleep states were described as N-REM and REM sleep; no details defining the two sleep states.	Apnoeas followed a sigh in 16 of 106 recorded during REM sleep and in 29 of 43 recorded during N-REM. 99 of the apnoeas were mixed/ obstructive (33 in NREM and 66 REM sleep). A significant suppression of EEG activity (p < 0.05) was found during mixed/obstructive N-REM apnoeas and inactive REM apnoeas. A significant EEG-suppression in the 8–13 Hz band in N-REM-sleep apnoeas with an initial sigh occurred (p < 0.05) in contrast to those without. Apnoea with bradycardia was observed during 11 REM in contrast to 13 N-REM.	This study is a very brief report as a conference abstract therefore many details were unclear. In particular the EEG recording techniques and analysis methods were not described. The results were not split by age at recording.
Holthausen et al. (1999)	Germany, Europe.	Cross-sectional.	Term and preterm (28–100 weeks GA).	71	Within-subject; before during and after apnoea.	Apnoea defined as a flat respiratory tracing for ≥ 3 s on both nasal airflow and thoracic movement monitors. No heart rate and oxygen saturation measurements were described.	Montage: 1 channel bipolar cEEG (C3-C4) Outcome measures: Mean power, power variance and entropy in 7 frequency bands: sub delta (0 ± 1.5 Hz), delta (1.5 ± 3.5 Hz), theta (3.5 ± 7.5 Hz), alpha (7.5 ± 13.5 Hz), beta 1 (13.5 ± 19.5 Hz), beta 2 (19.5 ± 25 Hz) and gamma (25 ± 50 Hz) using fast Fourier Transformation. EEG changes relative to sleep states were not evaluated.	Subjects were split into three groups: up to 35 weeks, 36 ± 40 weeks > 41–100 weeks GA. A significant reduction in normalised delta and theta amplitude during apnoeas compared with before and after apnoea was observed in infants > 41 weeks. Similar, but not statistically significant, changes were observed in the other two age groups	Inclusion and exclusion criteria were not clearly defined, and infant demographics were not presented. The main aim of the paper was to create a model that could predict the infant age category from the EEG but changes in amplitude during apnoea were examined. Methods, in particular, statistical methods, were not clearly defined.
Curzi-Dascalova et al. (2000)	France, Europe.	Cross-sectional.	Preterm (32–34 weeks GA, 2–15 days PNA).	5	Within-subject; 1 min before the onset of apnoea and of similar duration as the resultant apnoeic event.	Apnoea: reduction in abdominal respiratory movements amplitude by ≥ 20 % and/or airflow measured using strain gauges and thermistors, respectively, and lasting > 5 s duration. R-R intervals on ECG and cardiotocography tracings were recorded to assess heart rate changes. Pulse oximetry was also monitored and compared 1 sec before to 10 secs after an apnoea episode.	Montage: 3 channels bipolar cEEG with at least centro-occipital (C3-O1, C4-O2). Outcome measures: Amplitude changes (visually assessed) and frequency changes (assessed both visually and using a computer). Behavioural stages were described visually (using 30-second epochs) as wakefulness (presence of continuous movement or crying; eyes open with or without eye movement), active sleep (continuous EEG pattern, eye movements), quiet sleep (discontinuous EEG, no eye movement) and indeterminate sleep (discrepancy between active and quiet sleep criteria).	492 apnoeas were recorded and 27 were longer than 10 s. None of the apnoeas were preceded or followed by wakefulness. Only 77 (all during active or indeterminate sleep) were assessed for EEG changes. Others were excluded as they occurred close to other apnoeas, body movements or sighs. Most (62.7 %) apnoeas were not associated with a change in EEG amplitude and there was no significant difference from control periods. Using quantitative analysis, post-apnoea frequency changes did not occur in 10.4 % of apnoeas, 44.2 % of apnoeas showed an increase in frequency and 45.4 % showed a decrease. Although the proportion of apnoeas showing a frequency increase was similar to the control periods, the value of the change observed following an apnoea was significantly different (p < 0.02). Regression analysis showed that normalized percentage of EEG change was significantly dependent on apnoea type (obstructive versus central apnoeas (p < 0.007), with no change after central apnoea); amplitude modification (p < 0.008), and basal EEG frequency (p < 0.02). Frequency change was not related to apnoea duration or sleep state. Most apnoeas (58.9 %) were followed by bradycardia (pre and post apnoea difference p < 0.002), 34.7 % by tachycardia and no heart rate changes in 6.7 %. For control periods, bradycardia (50.6 %) tachycardia (42.9 %) and unchanged (6.5 %) were observed. Mean bradycardia following apnoea was significantly greater compared with control periods (p < 0.03) and were dependent on apnoea duration (p < 0.0001), baseline heart rate (p < 0.0007) but not on sleep state. Nearly half the apnoeas (48 %) were followed by desaturation, 17.3 % by an increase in saturation (this occurred when the baseline saturation was < 90 %) and no change during 37.7 % of apnoea episodes. There were no significant differences between the saturation changes following apnoea and control periods. There was no correlation between EEG frequency changes and changes in heart rate or oxygen saturation.	Definition of apnoea used is not standard (quite short), and with a very small sample size, making results difficult to interpret. The study is well described, with clear explanation of the subject inclusion criteria, methods of data collection and analysis.
Schramm et al. (2000)	Germany, Europe.	Cross-sectional.	Term and preterm (22–40 weeks GA, mean 34.7 weeks GA, mean 45.2 weeks PMA).	51	Within-subject; consisting of periods just before and after apnoea; and other apnoea-free phases of active sleep.	Not specified. ECG and pulse oximetry were recorded	Montage: Two channels bipolar cEEG (C3-T3, C4-T4). Outcome measures: Absolute power, relative power, median frequency, and peak frequency for sub delta (0.4 ± 1.5 Hz), delta (1.5 ± 3.5 Hz), theta (3.5 ± 7.5 Hz), alpha (7.5 ± 12.5 Hz), beta 1 (12.5 ± 19.5 Hz), and beta 2 (19.5 ± 25.0 Hz) band using spectral analysis. Sleep states were automatically annotated using a polysomnographic device.	A significant reduction in the mean and standard deviation of absolute EEG power during apnoeas in the sub delta, delta, theta and alpha frequency bands compared to before and after periods of apnoea and the apnoea-free phases of active sleep was observed. In the beta 1 band, significantly lower absolute power during the apnoea compared with before apnoea and the apnoea-free periods occurred (not different to after the apnoea). No significant power differences in the beta 2 band were observed. The highest relative reduction of 45 % was in the theta band. There were no differences in the means and standard deviations of peak frequencies in all the frequency bands.	No clear description of the exposure variable (e.g. duration of apnoea not specified). It is not clear what statistical test was used or whether all apnoeas were recorded in one study infant or more evenly distributed across participants. Infant demographic information was not clearly described. However, outcome measures were well described. No heart rate or saturation changes were described as part of the results. Sleep state comparison was not detailed, only active sleep state during apnoea free periods was considered.
Low et al. (2012)	Ireland, Europe.	Case report.	Term (32 weeks GA/38 weeks corrected GA).	1	Within subject; before, during and after the onset of apnoea.	Apnoea: >20 s, or < 20 s with associated bradycardia (20 % below baseline heart rate) or oxygen desaturation (<80 %) and measured with neonatal monitors. ECG and oxygen saturation were recorded simultaneously using a vital signs monitor.	Montage: 4 channels bipolar cEEG (F4-C4, F3-C3, C4-O2, C3-O1). Outcome measures: EEG suppression defined as amplitude reduction below 5 μV in all EEG channels for at least 10 s. This was reviewed and annotated by a neurophysiologist. Sleep states were not described.	8 apnoeas were recorded. 6 apnoeas (range 23–119 s) had no EEG changes with a mean lowest desaturation of 45 % and bradycardia 99 beats per minute. Two episodes (213 and 376 s) required resuscitation and were associated with a burst suppression pattern. These were preceded by a rapid drop in heart rate and saturation below 20 % with accompanying cyanosis. During recovery, EEG activity returned with improved oxygen saturation above 30 to 40 %.	A clearly described case report.
Manas et al. (2014)	Spain, Europe.	Cross-sectional- conference abstract.	Preterm (31–35 weeks corrected GA).	12	Within subjects; apnoea compared with period of normal respiration.	Not defined. Vital signs monitoring not specified.	Montage: 8 electrode cEEG (FP1, FP2, C3, C4, T3, T4, O1, O2) referenced to mastoids. Outcome measures: Functional connectivity assessed using the MSC and PSI in the delta frequency band (0.5–4 Hz) and NLGSI between all pairs of EEG channels were quantitatively evaluated. Active and quiet sleep states were recognised but definitions not provided.	MSC increased during quiet sleep apnoea compared to normal respiration in the same state (p < 0.001). Intra-hemispheric EEG channel pairs exhibited significant differences between normal respiration and apnoea (p < 0.001) which was dependent on sleep state (p < 0.01), whereas inter-hemispheric EEG channels showed differences regardless of the type of sleep (p < 0.01). NLGSI was greater for apnoea than during normal respiration (p < 0.01) irrespective of the sleep state. The PSI during normal respiration was not different from zero while during apnoea, it was significantly different from zero and positive (p < 0.01)	This is a conference abstract so limited details were given. In particular, the definition of apnoea was not given. The EEG measures used are not as commonly used as those used in some studies.

Abbreviations: ECG – electrocardiography; cEEG - conventional electroencephalography; EOG – electrooculogram; GA - gestational age; MSC - magnitude squared coherence function; NLGSI - nonlinear generalized synchronization index; NREM - non rapid eye movement; PSI - phase synchronization index; PMA - postmenstrual age; PNA - postnatal age; REM -rapid eye movement.

**Table 2:** Summary of included studies reporting on the effect of other respiratory changes on neonatal EEG.

**Author (year)**	**Country and WHO Region**	**Study design**	**Infant category and age**	**Sample size**	**Comparison group**	**Respiratory and cardiovascular change definition**	**EEG methods**	**Results**	**Quality assessment/Comments**
Whitehead et al. (2019)	United Kingdom, Europe.	Cross-sectional.	Term and preterm (23–41 weeks GA, 30–42 weeks PMA, 2–78 days PNA).	13: 10 met the review inclusion criteria (3 infants were excluded − 2 had germinal matrix intraventricular haemorrhage, 1 infant had ventriculomegaly)	Within-subject event-free period.	Hiccups: diaphragm contractions recorded with a movement transducer attached to the trunk. ECG was recorded and heart rate compared before and after bouts of hiccups. Oxygen saturation was observed at the cot side in 10 out of 13 infants.	Montage: 16–18 channel referential cEEG (F7, F8, F3, F4, Cz, C3, C4, T7, T8, P7, P8, O1, O2, CPz, CP3, CP4, TP9, TP10) with reference at Fz, re-referenced to common average for analysis. Outcome measures: ERP were quantitatively assessed using grand averages, individual subject traces and scalp maps. Both wakefulness and active sleep were characterised by movement, irregular breathing, and continuous low voltage EEG while quiet sleep was defined by the absence of movement, regular respiration, and a fluctuating EEG amplitude.	A total of 946 hiccups were recorded (in 10 infants that met this review’s inclusion criteria). Hiccups were more likely to occur in infants who were awake – 7 infants were awake at the start of hiccups while 3 were in active sleep. Hiccups elicited 3 distinct ERPs compared to baseline. ERP1 comprised of fronto-central-temporal negativity, with positivity most prominent across the posterior region at −49 to 35 ms (GFP peak latency: 16 ms). ERP2 showed central and posterior negativity, with positivity most prominent across the anterior and bi-temporal regions at 91 to 150 ms (GFP peak latency: 125 ms), while ERP3 showed central and posterior positivity, with negativity most prominent bi-temporally at 223 to 913 ms (GFP peak latency: 310 ms). There was no correlation between the ERP and infant corrected gestational age. There was no difference in heart rate and oxygen saturation before and after hiccup bouts.	Exploratory study identifying infants from an EEG research database who had hiccups during the recording. Methods clearly described.

Abbreviations: ECG – electrocardiography; EMG - electromyography; ERP - event-related potential; GA - gestational age; GFP - Global Field Power; NREM - non-rapid eye movement; PMA - postmenstrual age; PNA - postnatal age.

**Table 3:** Summary of included studies reporting the effect of respiratory stimulants used for apnoea on neonatal EEG features.

**Author (year)**	**Country and WHO Region**	**Study design**	**Infant category**	**Sample size**	**Exposure and comparison groups**	**Respiratory Stimulant and vitals measurement**	**EEG methods**	**Results**	**Quality assessment score**
Supcun et al. (2010)	Germany, Europe.	Cohort.	Preterm (<34 weeks GA, median GA 29 weeks (range, 24–33 weeks); 2 (range 1–14) days PNA).	51: 20 intubated	Within-subject comparison; preterm infants scheduled to receive a first dose of caffeine for prophylaxis or treatment of apnoea were compared from 2 h before until 2 h after administration of a loading dose of caffeine. Apnoea: oxygen saturation < 80 % for at least 5 s; assessment method unspecified.	Caffeine, intravenous loading dose 10 mg/kg body weight. Heart rate and arterial oxygen saturation were recorded. Details about methods of measurement not described.	Montage: 2 channels bipolar aEEG (C3-P3 and C4- P4). Outcome measures: EEG continuity assessed visually as continuous (minimum amplitude > 5 µV, maximum amplitude 10–50 µV) or discontinuous (minimum amplitude variable but < 5 µV, maximum amplitude > 10 µV) in 10-minute epochs. Quantitatively, one minute average values for the maximum, minimum, and mean amplitudes were calculated. No sleep staging was considered	After caffeine administration there was significant increase in the percentage of aEEG continuity (p < 0.001), and the maximum (p < 0.001), minimum (p < 0.001) and mean (p < 0.001) amplitudes compared to before caffeine use. These changes persisted throughout the 2-hour recording after caffeine administration, with no differences between the first 30 min after caffeine administration and the 30 min at the end of the 2-hour window. There were no significant changes in heart rate or oxygen saturation pre and post caffeine administration. In the 31 spontaneously breathing infants there was no difference in the number of apnoeic episodes between the 2 observation periods.	Very brief report, exploratory study for limited time range, but EEG measures described in detail.
Hassanein et al. (2015)	Egypt, Eastern Mediterranean.	Cohort.	Preterm (<34 weeks GA).	33: only 20 cases relevant to the review, 13 in the control group not relevant to the review and were only studied for follow-up comparison.	Within-subject comparison; Caffeine group receiving first dose of caffeine for prophylaxis or treatment of apnoea (recorded for 1 h before, during and 2 h after caffeine administration. Apnoea definition and measurement method not specified.	Caffeine citrate, intravenous loading dose 20 mg/kgbody weight. Continuous cardiovascular monitoring was performed but methods not specified.	Montage: Single aEEG biparietal channel recorded to assess changes in response to caffeine and 9 channel cEEG (Fp1, Fp2, C3, C4, T3, T4, O1, O2, and Cz, reference not given) recorded at 36 weeks. Outcome measures: aEEG continuity were assessed visually and defined as continuous with minimum amplitude > 5 µV and maximum amplitude of 10–50 µV; or discontinuous with variable minimum amplitude < 5 µV and maximum amplitude > 10 µV). cEEG was used to identify seizures and sleep arousal if they occurred. Sleep state differentiation into 6 stages (quiet, active, drowsy, quiet alert; active alert, crying) was considered but findings not described based on the classification.	There was a statistically significant increase in aEEG continuity 30 min after caffeine administration (51.96 % ± 34.30 %) compared to before (33.33 % ± 30.05 %, p = 0.002) in the caffeine treated group. Arousability significantly increased after caffeine administration, with more babies showing EEG features indicative of alertness compared with sleep (p < 0.001). No significant difference in apnoea occurrence with caffeine administration. Caffeine significantly increased heart rate (p = 0.0001), mean arterial blood pressure (p = 0.0001) and oxygen saturation (p = 0.003) compared with period before administration. At 36 weeks, aEEG score was significantly higher in the caffeine group (10.11 ± 1.62) compared to the control group (6.85 ± 1.77, p < 0.001), whereas conventional EEG background cerebral activity grade and electrographic seizure activity showed no significant difference between the two groups (p = 0.091, 0.38).	This study compared infants given caffeine for clinical reasons with those born at a similar age who were not given caffeine, either as it was not clinically necessary or because parents did not consent to caffeine treatment. There may be differences between the two groups. Half of the caffeine group were lost to follow up at 36 weeks but reasons are not given.
Dix et al. (2018)	The Netherlands, Europe.	Cohort.	Preterm (<32 weeks GA).	32	Within subject comparison; infants receiving their first dose of caffeine therapy prescribed by the treating neonatologist, measures compared before caffeine therapy and in the six hours after therapy started. Physiological parameters not defined but measured using a patient monitor.	Caffeine-base intravenous loading dose 10 mg/kg body weight. Patient monitors were used for physiology monitoring.	Montage: 2 channels aEEG, electrode positions not specified. Outcomes: SAT rate and ISI were calculated from the raw EEG; minimum, mean, and maximum amplitudes of aEEG. Sleep staging was not described.	There was a significant increase in SAT rate by 0.33/min per week GA (p < 0.001), and a decrease in ISI length of 0.42 s per week (p < 0.001) but no change with caffeine intake. Maximum, mean, and minimum amplitude did not change significantly after caffeine intake but were all significantly associated with GA. There was no significant difference in respiratory rate and oxygen saturation following caffeine administration but heart rate increased.	EEG results were not reported in detail e.g. for SAT rate and ISI length before compared with after caffeine start no values were stated or plots given. The methods for analysis are not clearly specified, such as how SATS were identified.
Yang et al. (2019)	China, Western Pacific	RCT.	Low birth weight preterm infants enrolled in either the caffeine group (mean 32.56 ± 2.35 weeks GA) and the control group who received aminophylline (33.02 ± 1.98 weeks GA).	212 (106 per group).	Between subject comparison; infants with apnoea treated with caffeine citrate were compared with controls that received aminophylline. Recording time frames were not indicated by authors. Apnoea definition and measurement not defined.	Caffeine citrate (intravenous loading dose 20 mg/kg, then maintenance dose 5 mg/kg every 24 h) or aminophylline (intravenous loading dose 10 mg/kg, then maintenance dose 2 mg/kg every 12 h). No physiology monitoring was reported.	Montage: aEEG electrode positions not specified. Outcomes: Sleep arousal cycle, graphic continuity, lower edge amplitude value, aEEG continuity voltage, periodic occurrence rate narrowband voltage, and bandwidth were evaluated using defined scoring systems. Quantification method was not specified. No sleep state categorisation was reported.	There were no significant differences in all the EEG outcomes between the caffeine and aminophylline groups before treatment. Sleep arousal cycle (p = 0.029), graphic continuity (p = 0.017), lower edge amplitude value (p = 0.047), continuous voltage positive rate (p = 0.011), sleep-wake cycle (p = 0.042), and lower boundary voltage (p = 0.007) were significantly higher in the caffeine group than in the aminophylline group. All measures increased in both groups after treatment compared with before, but no statistical analysis was performed (as the main outcome of the trial was the comparison between groups). aEEG detection bandwidth and narrow-band upper boundary voltage decreased after treatment, with significantly lower values in the caffeine group (p = 0.020, 0.032 respectively).	Many details were not reported in the paper, specifically relevant for this review for example, the time points at which the EEG measurements were taken, the equipment used, and the definition of apnoea. Treatment allocation concealment, blinding (treatment and outcome assessment), method used (reliability) for outcome assessment and deviations from standard RCT design were not specified. No correction for multiple comparisons was used and no measure was pre-selected as the primary outcome.

Abbreviations: aEEG – amplitude-integrated electroencephalography; GA - gestational age; ISI - inter SAT interval; PMA - postmenstrual age; PNA - postnatal age; RCT - randomised controlled trial; SATs - spontaneous activity transients.

### 3.1 Studies reporting on changes in EEG signals during periods of acute respiratory events

#### 3.1.1 Effect of apnoea on EEG brain activity

Nine of 14 (64.3 %) articles described the effect of apnoea on brain activity. All were observational studies with a combined sample size of 209 infants; over half (n = 5/9, 55.6 %) of the studies assessed both term and preterm infants (Table 1). All studies had a within-subject control, comparing EEG epochs during periods of normal breathing with apnoeic episodes. The definitions used for the exposure variable (apnoea), where stated, were diverse. Apnoea was defined in some articles to be as short as 3 s in duration, whereas other articles defined such periods as respiratory pauses, and apnoea as a pause of at least 20 s (Table 1).

The EEG features assessed varied, comprising of occurrence of burst suppression pattern (Deuel, 1973, Low et al., 2012, Wulbrand and Bentele, 1994), changes in continuity (Deuel, 1973), amplitude (Bridgers et al., 1985, Curzi-Dascalova et al., 2000, Fenichel et al., 1980, Holthausen et al., 1999), frequencies (Curzi-Dascalova et al., 2000, Schramm et al., 2000), absolute and relative power (Holthausen et al., 1999, Schramm et al., 2000), magnitude squared coherence function, phase synchronization and nonlinear generalized synchronization (Manas et al., 2014). All but three studies (Bridgers et al., 1985, Holthausen et al., 1999, Low et al., 2012) described sleep staging as part of EEG evaluation. Further, the method of assessment varied, with some (n = 3/9, 33.3 %) studies reporting EEG features evaluated through visual assessment from experts only (Bridgers et al., 1985, Fenichel et al., 1980, Low et al., 2012) and some (n = 3/9, 33.3 %) using quantitative assessment (Holthausen et al., 1999, Manas et al., 2014, Schramm et al., 2000). One study (Curzi-Dascalova et al., 2000) used both visual assessment and quantitative analysis and generally reported similar results with both, though statistically significant changes were only observed in the quantitative analysis. While most studies used statistical analysis (Curzi-Dascalova et al., 2000, Holthausen et al., 1999, Manas et al., 2014, Schramm et al., 2000, Wulbrand and Bentele, 1994), other studies did not give the frequency of occurrence of the EEG changes they observed (Bridgers et al., 1985, Deuel, 1973), instead reporting that they occurred ‘frequently’ or ‘sometimes’ (Table 1).

Of the 9 studies, simultaneous apnoea and EEG suppression were reported in three articles (one of which is a conference abstract) (Deuel, 1973, Low et al., 2012, Wulbrand and Bentele, 1994), a generalised reduction in EEG amplitude was reported in two studies (Bridgers et al., 1985, Fenichel et al., 1980), and a reduction in the mean and standard deviation of absolute EEG power in one study (Schramm et al., 2000). Holthausen et al. (1999) reported a decrease in amplitude only in the delta and theta bands. One study showed a significant post-apnoea frequency change, with some infants having an increase – while others a decrease – in frequency compared with the control period one minute before the apnoea (Curzi-Dascalova et al., 2000). Significant increases in the magnitude squared coherence, and nonlinear generalized synchronization index during apnoea relative to control periods were also reported (as a conference abstract) (Manas et al., 2014).

In contrast, no change in EEG brain activity was observed during most episodes of apnoea studied by Fenichel et al. (1980) (n = 28/35 apnoeas, 80 %) and Low et al. (2012) (n = 6/8 apnoeas, 75 %). Similarly, no change in amplitude in some apnoeas (proportion not provided by authors) was reported by Bridgers et al. (1985) and in 62.7 % of apnoeas observed by Curzi-Dascalova et al. (2000). Only one study (Holthausen et al., 1999) compared preterm infants based on age and reported a significant reduction in theta and delta amplitude during apnoea in term infants ≥ 41 weeks; this change was not significant in younger infants.

#### 3.1.2 Effect of other respiratory changes on EEG

One of the 14 studies (7.1 %) reported respiratory changes other than apnoea, describing EEG-recorded evoked responses to hiccups (Whitehead et al., 2019) (Table 2). This was a cross-sectional study involving 13 term and preterm infants (10 of whom met the inclusion criteria for this review). The authors identified three distinct hiccup-related potentials with peaks occurring at 16, 125 and 310 ms after contraction of the diaphragm, predominantly in central regions.

### 3.2 Relationship between characteristics of acute respiratory events and brain activity

Three studies (Deuel, 1973, Fenichel et al., 1980, Low et al., 2012) evaluated EEG changes relative to apnoea severity; all reported EEG amplitude decreases and burst suppression during some, but not all, long apnoeas, and, in some cases, in shorter apnoeas. The description of apnoea duration in the studies was non-uniform. Fenichel et al. (1980) defined long (≥20 s) and short (≤19 s) apnoeas, and reported 6 of 19 short apnoeas had mild amplitude suppression, and amplitude suppression only during 1 out of 16 long apnoeas. Deuel (1973) reported burst suppression at the start of respiratory pauses and apnoeas (Table 1). Low et al. (2012) found in one infant (case report) that changes in oxygen saturation below 20 % during two episodes of prolonged apnoea requiring resuscitation were associated with complete EEG burst suppression; this was not observed in shorter episodes of apnoea in the same infant.

Most (n = 7/9, 77.8 %) of the studies simultaneously recorded heart rate and/or oxygen saturation with EEG monitoring (Table 1), however, two of these studies (Schramm et al., 2000, Bridgers et al., 1985) did not describe the vital signs changes in relation to brain activity changes. Fenichel et al. (1980) did not observe a consistent relationship with heart rate changes and EEG suppression and Curzi-Dascalova et al. (2000) reported no correlation between changes in EEG frequency during apnoea and changes in heart rate or oxygen saturation (Table 1). Deuel (1973) reported that EEG suppression occurred simultaneously with apnoea, whereas bradycardia occurred approximately 15 s later in the single infant that met this review’s inclusion criteria (Table 1).

Curzi-Dascalova et al. (2000) found that changes in EEG frequency were dependent on apnoea type (with greater EEG changes following obstructive apnoea compared with central apnoea), amplitude modification and baseline EEG frequency in their study of five infants, but frequency changes were not related to apnoea duration or sleep state.

### 3.3 Studies reporting on the effect of respiratory stimulants on EEG

Four of 14 (28.6 %) of the included studies described the effect of respiratory stimulants (used for apnoea treatment or given prophylactically) on the EEG in a total of 315 infants **(**Table 3**)**. Caffeine in varying doses was used in all four studies (Table 3), and aminophylline use was reported in one study (Yang et al., 2019). All four studies were of preterm infants, one was an RCT (Yang et al., 2019) and three were cohort studies (Dix et al., 2018, Hassanein et al., 2015, Supcun et al., 2010).

The studies summarised EEG changes before, during, and after the use of respiratory stimulants, and all used amplitude-integrated EEG. None of the included studies found a significant difference in the apnoea rate or respiratory rate after stimulant introduction compared with before, however, in many cases the stimulant was given prophylactically. The aEEG features assessed included changes in continuity pattern, arousal from sleep (Hassanein et al., 2015, Yang et al., 2019), spontaneous activity transient (SAT) rate and interval, (Dix et al., 2018), aEEG amplitude (Dix et al., 2018, Supcun et al., 2010, Yang et al., 2019), voltage and bandwidth (Yang et al., 2019). Importantly, no respiratory stimulant studies investigated how changes in EEG during apnoea were altered with stimulant use – they only investigated EEG changes in relation to the start of drug therapy. They also did not explore simultaneous interactions between EEG and changes in heart rate, oxygen saturation or respiration.

Respiratory stimulant use significantly increased aEEG continuity (Hassanein et al., 2015, Supcun et al., 2010, Yang et al., 2019), sleep arousal (Hassanein et al., 2015, Yang et al., 2019), SAT rate (Dix et al., 2018), lower edge amplitude and boundary voltage values (Yang et al., 2019). Dix et al. (2018), however, reported no changes in EEG amplitude after caffeine administration. The SAT interval (Dix et al., 2018) bandwidth and narrow-band upper boundary voltage (Yang et al., 2019) were reduced following respiratory stimulant administration. Yang et al. (2019) found that EEG changes were greater with caffeine than with aminophylline use.

### 3.4 Quality assessment of the included articles

Comments on quality assessment for each of the included studies are summarised in Table 1, Table 2, Table 3 and the results of the JBI checklists are given in Appendix B. Three papers (Curzi-Dascalova et al., 2000, Low et al., 2012, Whitehead et al., 2019) were very well described and clear in terms of subjects, exposure and outcome measures. Other studies lacked detail in one or more key areas, such as subject inclusion criteria, methodology related to respiratory measurement, EEG measurement or analysis. Two papers (Manas et al., 2014, Wulbrand and Bentele, 1994) were conference abstracts and so details were very limited; we chose to include these studies as details were sufficient for inclusion, however, it should be noted that these were not full articles. Several studies (Bridgers et al., 1985, Deuel, 1973, Fenichel et al., 1980) used visual EEG assessment (in part as they were from the 1980s or before) – this is the clinical gold standard, nevertheless, it is subjective and may lack reproducibility. Statistical analysis was lacking in several studies, and where statistical analysis was used, most papers did not appear to correct for multiple comparisons.

## 4 Discussion

This systematic review aimed to investigate the effect of both acute respiratory events and respiratory stimulants on brain activity recorded using EEG in neonates. We identified 14 studies conducted up until August 2022, involving a relatively small number of infants. Nine studies investigated EEG changes in relation to episodes of apnoea. Neonatal apnoea-related EEG changes were inconsistent, with EEG suppression, amplitude and post-apnoea frequency reduction observed during some, but not all, episodes of apnoea. The factors that drive these differences are yet to be elucidated, with differences across studies in definition of apnoea, and other changes in vital signs (e.g., bradycardia and desaturation), making comparison across studies difficult. One study investigated other respiratory events – characterising evoked cortical responses to hiccups, observed in both preterm and term infants. Four studies investigated EEG changes in relation to respiratory stimulants, demonstrating increased EEG continuity, arousability, lower edge amplitude and boundary voltage values and decreased SAT interval, bandwidth and narrow-band upper boundary voltage.

EEG suppression, amplitude and frequency reduction during some apnoeic episodes suggests neuronal desynchrony with diffuse brain inactivity, most likely from cerebral anoxia (Shanker et al., 2021). Brain activity is critical in brain development (Ghosh and Shatz, 1992, Kanold et al., 2003, Ranasinghe et al., 2015, Tolner et al., 2012, Wikström et al., 2012) and apnoea has been associated with poor neurodevelopmental outcomes (Greene et al., 2014, Janvier et al., 2004, Pillekamp et al., 2007, Poets et al., 2015). Nevertheless, the extent to which apnoea contributes to (and is not just correlated with) poorer outcomes remains unclear (Erickson et al., 2021, Williamson et al., 2021). EEG suppression during apnoea may be neuroprotective to reduce cell energy consumption (Low et al., 2012), however, very long periods of oxygen deprivation, such as those in hypoxic-ischaemic brain injury, are associated with prolonged EEG suppression, which in turn is an indicator of poor outcome (Dereymaeker et al., 2019, Douglass et al., 2002). It is not known how long an apnoea can last in an infant without brain damage occurring (Low et al., 2012) or whether periods of EEG suppression during apnoea are related to neurodevelopmental outcomes. While Low et al. (2012) reported in a single infant, complete EEG suppression with extremely low oxygen level during a prolonged apnoea, Fenichel et al. (1980) did not observe a significant relationship between EEG changes and apnoea duration. The latter study also had a small sample size and the relationship with other factors such as the degree of oxygen desaturation and bradycardia (Pichler et al., 2003) which likely play an important synergistic role in brain function, were not clear. Curzi-Dascalova et al. (2000) found no correlation between EEG frequency changes and changes in heart rate or oxygen saturation. However, there is likely a complex interplay between factors such as apnoea duration, bradycardia, and oxygen desaturation with EEG changes. Further work in larger samples of infants is needed to understand these relationships.

EEG activity changes greatly with gestational age from a relatively discontinuous pattern in the extremely preterm infant to a more continuous pattern by term (André et al., 2010). Only one study considered EEG changes during apnoea in relation to the age of the infant, observing a significant amplitude reduction in infants > 41 weeks (Holthausen et al., 1999). In younger infants, similar trends were observed; the authors may have been underpowered to observe a significant effect in these age groups. To date, studies have not investigated if there is a relationship between the number of apnoeas an infant experiences and EEG changes, and it is unclear if the frequency of apnoea impacts brain function.

We only identified one study that investigated other acute respiratory events – Whitehead et al. (2019) identified event-related potentials in response to hiccups in both term and preterm infants. This demonstrates that contraction of respiratory muscles provides input to the brain, even in young preterm infants, which may drive the establishment of interoceptive circuits (Whitehead et al., 2019). Further studies in this area and those examining brain-respiratory interactions during periods of normal breathing are key to understanding the formation of brain-respiratory networks in preterm infants and factors that disrupt their development. Moreover, we did not identify any studies which examined other respiratory events, such as shallow breathing or periodic breathing. Further studies in this area will enable a comparison with findings on the effect of apnoea on brain function.

We included four studies that investigated the effects of respiratory stimulants on the EEG. We excluded three other papers investigating the effect of respiratory stimulants on the EEG – two which examined the effect of respiratory stimulant use on sleep states only (Curzi-Dascalova et al., 2002, Seppä-Moilanen et al., 2022) and one study which involved neonates with clinical seizures, cardiovascular, and CNS abnormalities (Czaba-Hnizdo et al., 2014). Of the included studies, one study – an RCT (Yang et al., 2019) – compared the effect of caffeine and aminophylline, while the other three (Dix et al., 2018, Hassanein et al., 2015, Supcun et al., 2010) studied the effect of caffeine only. The four studies did not specifically look at EEG changes during apnoea or in relation to respiration, rather, they evaluated the effect of methylxanthines on EEG activity. Although previous studies have reported decreased apnoea rate with the use of methylxanthines (Henderson-Smart and De Paoli, 2010), none of the included studies found a significant difference in the apnoea or respiratory rate after respiratory stimulant use compared with before, though this may be because in many cases stimulants were given prophylactically soon after birth. All four studies showed changes in the EEG with the use of respiratory stimulants, but further work is needed to ascertain whether and how these changes are related to other factors, such as age, dose, and comorbidities, and to understand how these changes in EEG might affect brain development. Understanding the effect of respiratory stimulants on brain activity may help drive individualised drug dosing in infants (Hartley, 2021, Hartley et al., 2021, Kumar et al., 2020).

This review was limited by the small number of studies, many of which had very small sample sizes. Two of the papers were conference abstracts with limited information (Manas et al., 2014, Wulbrand and Bentele, 1994), one was a case report (Low et al., 2012) which have known study design limitations (Nissen and Wynn, 2014), and three (Bridgers et al., 1985, Deuel, 1973, Fenichel et al., 1980) were published 40 to 50 years ago, and so findings may be obsolete with improvements in medical technology. There were diverse definitions for the exposure variable i.e., the definition of apnoea – with wide variation in minimum duration, different EEG outcome measures, methods of analysis (visual versus quantitative assessment) and methods of apnoea detection (which may also be unreliable in infants (Adjei et al., 2021)) making cross-study comparison challenging. The heterogeneity of studies also prevented a *meta*-analysis and power calculations with pooled data for future studies. Some studies used few EEG channels which can result in important loss of spatial information (Cherian et al., 2008, Ebersole et al., 1983). Further, the studies investigating respiratory stimulants used aEEG, which is less sensitive than cEEG (Falsaperla et al., 2022, Kadivar et al., 2019). To our knowledge, this is the first review summarising existing data on this topic. Our findings highlight the need for future large prospective research investigating the relationship between EEG and respiratory activity in infants.

## 5 Conclusion

Evaluating the relationship between brain activity and respiration in newborn infants is key to understanding the impact of apnoea on neurodevelopment and the potentially cyclical relationship between brain development and respiratory dynamics. Current studies in this area are limited by small sample sizes, and inconsistent definitions of apnoea and EEG methods make study comparisons challenging. Further research is needed to explore the impact of a multitude of factors, such as age, duration of apnoea and oxygen saturation on changes in brain activity. Current studies investigating the effect of respiratory stimulants on brain activity in infants are limited and have investigated the effect of start of treatment on brain activity, with no studies exploring brain activity changes during apnoea. Further research is needed to investigate how brain activity changes are related to factors such as drug dose and to gain a mechanistic understanding of how changes in brain activity with stimulant use impact on brain development. Understanding the effects of apnoea and optimising treatment options is essential to improving the long-term outcomes of premature infants.

## Declaration of competing interest

The authors declare that they have no known competing financial interests or personal relationships that could have appeared to influence the work reported in this paper.

## Figures and Tables

**Table:** 

**Variables assessed**	Low et al. (2012)
Were patient’s demographic characteristics clearly described?	Yes (excluding race)
Was the patient’s history clearly described and presented as a timeline?	Yes
Was the current clinical condition of the patient on presentation clearly described?	Yes
Were diagnostic tests or assessment methods and the results clearly described?	Yes
Was the intervention(s) or treatment procedure(s) clearly described?	Not applicable
Was the post-intervention clinical condition clearly described?	Not applicable
Were adverse events (harms) or unanticipated events identified and described?	Not applicable
Does the case report provide takeaway lessons?	Yes

**Table:** 

** Variables assessed**	Hassanein et al. (2015)	Dix et al. (2018)	Supcun et al. (2010)	Wulbrand and Bentele (1994)
Were the two groups similar and recruited from the same population?	Yes: Within subject comparison.	Yes: Within subject comparison.	Yes: Within subject comparison.	Yes: Within subject comparison.
Were the exposures measured similarly to assign people to both exposed and unexposed groups?	Not Applicable.	Not Applicable.	Not Applicable.	Not Applicable.
Was the exposure measured in a valid and reliable way?	Unclear: Apnoea measured clinically but assessment method and definition not specified. Caffeine given according to clinical practice.	Unclear: Physiological parameters measured using patient monitor, but no definition of apnoea given.	Unclear: Apnoea definition given, but assessment method not specified.	Yes: Airway occlusion, respiration measured using bands
Were confounding factors identified?	Yes: Exclusion criteria specified.	Yes: Age was considered.	Yes: Six infants excluded for artefacts or high EEG impedance.	No.
Were strategies to deal with confounding factors stated?	Yes.	Yes.	Yes.	No.
Were the groups/participants free of the outcome at the start of the study (or at the moment of exposure)?	Not applicable: Outcome measure is EEG change.	Not applicable: Outcome measure is EEG change.	Not applicable: Outcome measure is EEG change	Not applicable: Outcome measure is EEG change
Were the outcomes measured in a valid and reliable way?	Unclear: aEEG methods clearly described, however, EEG assessment was visual.	Yes.	Yes.	Yes.
Was the follow up time reported and sufficient to be long enough for outcomes to occur?	Unclear: Follow-up was for two hours after caffeine administration. Half-life of caffeine is much longer.	Unclear: Follow-up was for six hours after caffeine administration. Half-life of caffeine much longer.	Unclear: Follow-up for two hours after caffeine administration. Half-life of caffeine much longer.	Yes.
Was follow up complete, and if not, were the reasons of loss to follow up described and explored?	Yes: Loss to follow up of caffeine group at 36 weeks not clearly reported, but this was not relevant for our review.	Yes.	Yes.	Yes.
Were strategies to address incomplete follow up utilized?	Not applicable.	Not applicable	Not applicable.	Not applicable.
Was appropriate statistical analysis used?	Yes, but no adjustment for multiple comparisons.	Yes, but no adjustment for multiple comparisons.	Yes, but no adjustment for multiple comparisons.	Yes.

**Table:** 

** Variables assessed**	Deuel (1973)	Fenichel et al. (1980)	Bridgers et al. (1985)	Holthausen et al. (1999)	Curzi-Dascalova et al. (2000)	Schramm et al. (2000)	Mañas et al. (2014)	Wulbrand et al. (1998)	Whitehead et al. (2019)
Were the criteria for inclusion in the sample clearly defined?	No.	Yes.	Yes.	No: Inclusion and exclusion criteria not specified	Yes.	Yes.	No: Conference abstract; many details were unreported including subject inclusion and exclusion criteria	No: Conference abstract; many details, including subject inclusion and exclusion criteria were not documented.	Yes.
Were the study subjects and the setting described in detail?	Unclear: No detailed description of background and medical history.	Yes.	Unclear: Demographic information not detailed	No: No detailed subject description.	Yes	Unclear: Limited information given.	Unclear: Limited detail given	Unclear: Limited detail given.	Yes.
Was the exposure measured in a valid and reliable way?	Yes.	Yes: Apnoea definition not standard but duration described as 4 seconds. Assessment method was clear.	Yes.	Yes.	Yes.	Unclear: Device name given and it automatically detects events, but detection algorithm unclear. No detail on average number of events per infant or whether all infants experienced apnoeas.	Unclear: No detail given on recording method or definition of apnoea.	Yes.	Yes.
Were objective, standard criteria used for measurement of the condition?	Yes.	Unclear: Apnoea duration described as 4 seconds which is short and not standard definition. Measurement method was clearly defined.	Yes.	Yes.	Yes.	Unclear: Duration of apnoea not specified.	Unclear: No detail given on recording method or definition of apnoea.	Yes.	Yes.
Were confounding factors identified?	Yes: Identified seizures, noted in one infant.	Yes: Groups split according to convulsive and non-convulsive apnoeas clearly.	No: Seizure activity discussed but results not separated or adjusted	No.	Yes: Excluded apnoeas close to body movements.	No.	Yes: Sleep state investigated	Yes: Sleep state was investigated	Yes: Age and germinal matrix haemorrhage considered. Also discussed that findings unlikely to be caused by hiccup artefact on EEG.
Were strategies to deal with confounding factors stated?	No.	Yes: Subgroup reporting.	No.	No.	Yes.	No.	No, the authors state there was a difference with sleep state but do not present what the difference is.	Yes: Results were split according to sleep state, although detail of results is missing as this was an abstract.	Yes: Investigated single subject averages and the strength of the ERP was unrelated to age.
Were the outcomes measured in a valid and reliable way?	Unclear: EEG outcome assessed visually.	Unclear: Visual assessment of EEG.	Unclear: EEG visually assessed by 2 raters. Mainly focused on seizure detection rather than overall EEG changes.	Yes: But few details provided on EEG recording method.	Yes: EEG changes evaluated qualitatively by trained expert and using algorithm.	Yes: Outcome measures were clearly described.	Yes.	Unclear: No detail for EEG methods or how EEG suppression was defined.	Yes.
Was appropriate statistical analysis used?	No: Statistical analysis not conducted.	No: Statistical analysis not conducted.	No: Statistical analysis not conducted.	No: Authors stated findings as 'significant' in 1 group but did not indicate what statistical tests were used or the outcome of the test.	Yes: Statistics clearly reported.	Unclear: Statistical analysis with 'one-way analysis' performed but the exact test was not specified.	Unclear: Statistical test not specified.	Unclear: Appropriate statistics seem to have been used but unclear as little detail given of the measures assessed.	Yes.

**Table:** 

**Variables assessed**	Yang et al. (2019)
Was true randomization used for assignment of participants to treatment groups?	Unclear: Authors reported random table was method used, but further details were lacking.
Was allocation to treatment groups concealed?	No.
Were treatment groups similar at the baseline?	Yes.
Were participants blind to treatment assignment? *	Unclear: Participants were infants; unclear whether parents were blinded
Were those delivering treatment blind to treatment assignment?	No: No information mentioned about blinding.
Were outcomes assessors blind to treatment assignment?	No.
Were treatment groups treated identically other than the intervention of interest?	Yes: Control group received aminophylline instead of caffeine.
Was follow up complete and if not, were differences between groups in terms of their follow up adequately described and analysed?	Yes.
Were participants analysed in the groups to which they were randomized?	Unclear: Authors did not specify.
Were outcomes measured in the same way for treatment groups?	Yes.
Were outcomes measured in a reliable way?	Unclear: EEG outcome methods unclear, quantification method unclear, and authors did not specify when measures were taken, what equipment was used etc.
Was appropriate statistical analysis used?	Yes, but no correction for multiple comparisons or baseline adjustment
Was the trial design appropriate, and any deviations from the standard RCT design (individual randomization, parallel groups) accounted for in the conduct and analysis of the trial?	Unclear: Limited information described.

## Appendix A Database search strategies

### Step-by-step search strategy for Embase via Ovid

1 exp electroencephalography/ or (EEG or electroencephalogra* or EEG pattern or EEG maturation or EEG brain activity or brain activity or brain maturation or brain development or brain dysmaturity or brain wave* or background EEG or stimul* evok* EEG or interburst interval* or inter burst interval*).mp.
2 exp Infant, Newborn/ or (neonat* or newbor* or new-bor* or term or full term or full-term or fullterm or premat* or baby or babies or infant*).mp.
3 exp Respiration/ or (breath* or respirat* or apno* or apne* or breath* cess* or stop breath* or breath* hold* or period* breath* or inter breath interval* or interbreath interval*).mp.
4 1 and 2 and 3.
5 limit 4 to english language.
6 limit 5 to human.
7 limit 6 to infant <to one year>.

### Step-by-step search strategy for MEDLINE via Ovid

1 exp Electroencephalography/or (EEG or electroencephalogra* or brain activity or brain maturation or brain development or brain dysmaturity or brain wave* or interburst interval* or inter burst interval*).mp.
2 exp Infant, Newborn/or (neonat* or newbor* or new-bor* or term or full term or full-term or fullterm or premat* or baby or babies or infant*).mp.
3 exp Respiration/ or (breath* or respirat* or apno* or apne* or inter breath interval* or interbreath interval*).mp.
4 1 and 2 and 3.
5 limit 4 to (english language and humans).
6 limit 5 to “newborn infant (birth to 1 month)”.

### Step-by-step search strategy for PsycINFO via Ovid

1 exp electroencephalography/or (EEG or electroencephalogra* or EEG pattern or EEG maturation or EEG brain activity or brain activity or brain maturation or brain development or brain dysmaturity or brain wave* or background EEG or stimul* evok* EEG or interburst interval* or inter burst interval*).mp.
2 exp Infant, Newborn/or (neonat* or newbor* or new-bor* or term or full term or full-term or fullterm or premat* or baby or babies or infant*).mp.
3 exp Respiration/ or (breath* or respirat* or apno* or apne* or breath* cess* or stop breath* or breath* hold* or period* breath* or inter breath interval* or interbreath interval*).mp.
4 1 and 2 and 3.
5 limit 4 to english language.
6 limit 5 to human.
7 limit 6 to 120 neonatal <birth to age 1 mo>.

### Step-by-step search strategy for Global health via Ovid

1 exp electroencephalography/or (EEG or electroencephalogra* or EEG pattern or EEG maturation or EEG brain activity or brain activity or brain maturation or brain development or brain dysmaturity or brain wave* or background EEG or stimul* evok* EEG or interburst interval* or inter burst interval*).mp.
2 exp Infant, Newborn/or (neonat* or newbor* or new-bor* or term or full term or full-term or fullterm or premat* or baby or babies or infant*).mp.
3 exp Respiration/ or (breath* or respirat* or apno* or apne* or breath* cess* or stop breath* or breath* hold* or period* breath* or inter breath interval* or interbreath interval*).mp.
4 1 and 2 and 3.
5 limit 4 to english language.

### Step-by-step search strategy for CINAHL via EBSCOhost

S1 (MH “Electroencephalography”) or EEG OR electroencephalogra* OR brain activity OR brain maturation OR brain development OR brain dysmaturity OR brain wave* OR interburst interval* OR inter burst interval*

S2 (MH “Infant, Newborn +”) or neonat* OR newbor* OR new-bor* OR term OR full term OR full-term OR fullterm OR premat* OR baby OR babies OR infant*

S3 (MH “Respiration+”) or breath* OR respirat* OR apno* OR apne* OR inter breath interval* OR interbreath interval*

S4 S1 and S2 and S3

S5 limit S4 to english

S6 limit S5 to infant, newborn: birth-1 month

### Step-by-step search strategy for Web of Science

1 1 TS=(EEG OR electroencephalogra* OR brain activity OR brain maturation OR brain development OR brain dysmaturity OR brain wave* OR interburst interval* OR inter burst interval* )
2 TS=(neonat* OR newbor* OR new-bor* OR term OR full term OR full-term OR fullterm OR premat* OR baby OR babies OR infant*)
3 TS=(breath* OR respirat* OR apno* OR apne* OR inter breath interval* OR interbreath interval*)
4 1 and 2 and 3 and English

### Step-by-step search strategy for ProQuest

((EEG OR electroencephalogra* OR 'brain activity' OR 'brain maturation' OR 'brain development' OR 'brain dysmaturity' OR 'brain wave*' OR 'interburst interval*' OR 'inter burst interval*') AND (neonat* OR newbor* OR new-bor* OR term OR 'full term' OR full-term OR fullterm OR premat* OR baby OR babies OR infant*) AND (breath* OR respirat* OR apno* OR apne* OR 'inter breath interval*' OR 'interbreath interval*'))

### Step-by-step search strategy for Cochrane Library

1 MeSH descriptor: [Electroencephalography] explode all trees
2 (EEG OR electroencephalogra* OR brain activity OR brain maturation OR brain development OR brain dysmaturity OR brain wave* OR interburst interval* OR inter burst interval*)
3 MeSH descriptor: [Infant, Newborn] explode all trees
4 (neonat* OR newbor* OR new-bor* OR term OR full term OR full-term OR fullterm OR premat* OR baby OR babies OR infant*)
5 MeSH descriptor: [Respiration] explode all trees
6 (breath* OR respirat* OR apno* OR apne* OR inter breath interval* OR interbreath interval*)
7 1 OR 2
8 3 OR 4
9 5 OR 6
10 7 AND 8 AND 9

## Appendix B JBI Checklist tables

Table B.1: JBI Critical Appraisal Checklist for the Included Case Report StudiesTable B.2: JBI Critical Appraisal Checklist for the Included Cohort StudiesTable B.3: JBI Critical Appraisal Checklist for Cross-sectional StudiesTable B.4: JBI Critical Appraisal Checklist for the Included Randomised Controlled Trials Studies
